# Supplementary material for: NAD+ repletion with niacin counteracts cancer cachexia
Source: Nat Commun. 2023 Apr 3;14:1849. doi: 10.1038/s41467-023-37595-6 (PMC10070388; doi:10.1038/s41467-023-37595-6)
Supplement: Supplementary file 1 — Supplementary Information [file 41467_2023_37595_MOESM1_ESM.pdf]

NAD+ REPLETION WITH NIACIN COUNTERACTS CANCER CACHEXIA

Supplementary Fig. 1

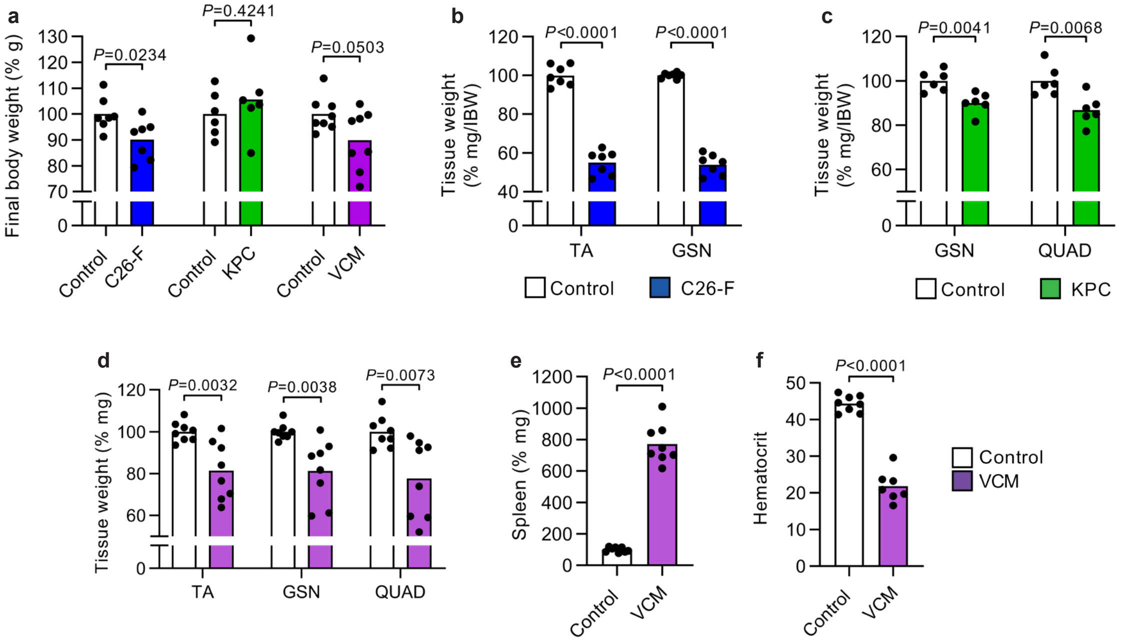

Supplementary Fig. 1. Body weight loss and muscle mass depletion in C26-F, KPC and VCM models

a Final body weight of C26-F (*n*=7 per group), KPC (*n*=6 per group) and VCM (*n*=8 per group) mice expressed as percentage of the mean of their respective control group. **b** Wet weight of *tibialis anterior* (TA) and *gastrocnemius* (GSN) muscles of C26-F mice normalized by initial body weight (IBW) and represented as a percentage of the mean of the control group (*n*=7 per group). **c** Wet weight of GSN and *quadriceps femoris* (QUAD) muscles of KPC mice normalized by IBW and represented as a percentage of the mean of the control group (*n*=6 per group). **d** Wet weight of TA, GSN and QUAD muscles represented as a percentage of the mean of the control group (*n*=8 per group). **e-f** Spleen wet weight (**e**) and hematocrit (**f**) of VCM mice represented as percentage of the mean of the control group (*n*=8 per group, except VCM hematocrit *n*=7). Data display: **a-f** are means with individual values. Statistical analysis was performed using two-tailed Student's t-test. Original raw data are provided as a Source Data file.

Supplementary Fig. 2

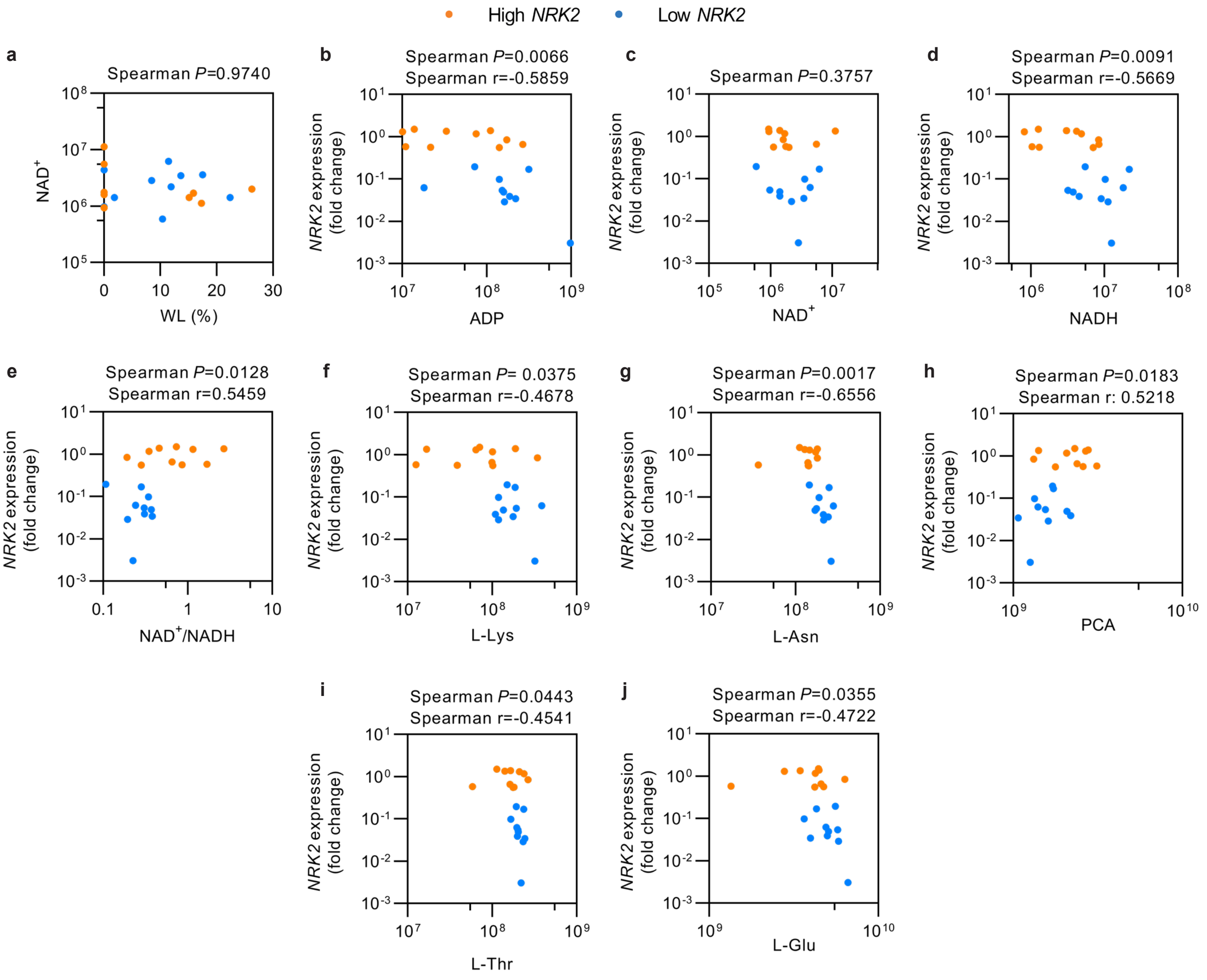

Supplementary Fig. 2. Correlation among NAD⁺ metabolism, body weight loss and metabolomic alterations in human CC

a Correlation plot between muscle NAD+ levels and percentage weight loss (WL %) of individual cancer patients. **b-j** Correlation plots between *NRK2* expression and particular metabolites in individual cancer patients (*n*=20). Statistical analysis was performed with two-tailed Spearman's correlation. Original raw data are provided as a Source Data file.

Supplementary Fig. 3

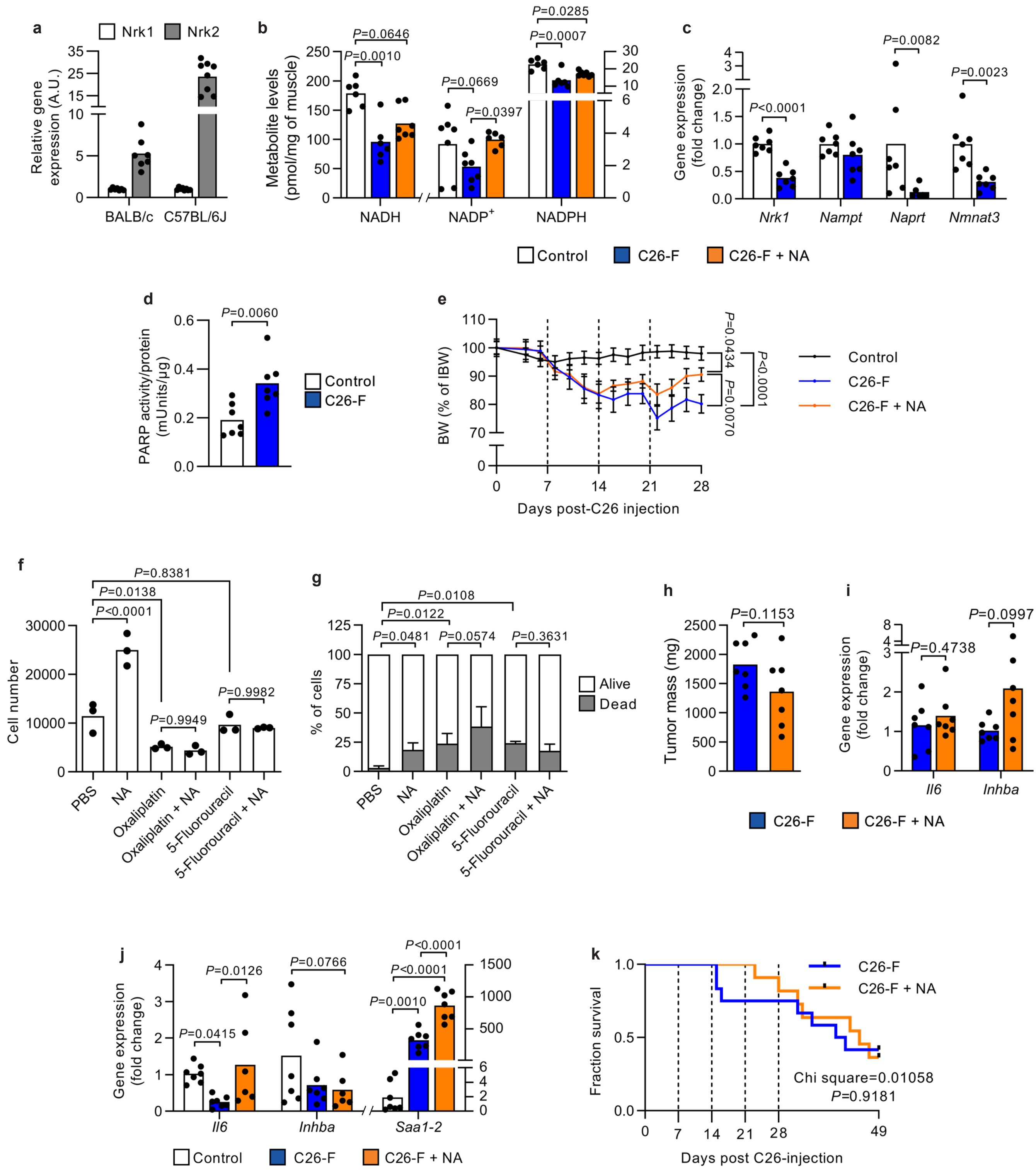

Supplementary Fig. 3. Characterization of niacin action on C26-F mice and C26 cells.

a Relative expression of *Nrk1* and *Nrk2* genes in the skeletal muscle of BALB/c (*n*=7 per gene) and C57BL/6J (*n*=8 per gene) mouse strains. **b** Levels of NADH, $NADP^+$ and NADPH represented as pmol per mg of muscle (*n*=7 per group, except control NADH *n*=6, C26-F NADH *n*=6, C26-F + NA $NADP^+$ *n*=6, control NADPH *n*=6, C26-F NADPH *n*=6). **c** Relative expression of genes involved in NAD^+ biosynthesis in the muscle of Control and C26-F groups (*n*=7 per group, except C26-F *Naprt n*=6). **d** Total PARP activity as mUnits normalized to total protein content (*n*=7 per group). **e** Body weight change of control, C26-F and C26-F + NA groups upon C26 tumor implantation (*n*=7 per group/experimental point). Dotted lines indicate Folfox treatment. Data are represented as percentage of mouse initial body weight (IBW). **f-g** Assessment of cell number (**f**) and death ratio of C26 cells (**g**) after 24h of exposure to 1mM NA, 10 µM oxaliplatin and 1 µM 5-fluorouracil alone, and both chemotherapeutics combined individually with NA (*n*=3 independent wells per group). **h** Tumor mass in C26-F and C26F + NA groups after tissue collection (*n*=7 per group). **i** Relative expression of *Il6* and *Inhba* genes in the tumors of C26-F and C26-F + NA groups (*n*=7 per group). **j** Relative gene expression of *Il6*, *Inhba* and *Saa1-2* genes in the liver of Control, C26-F and C26-F + NA groups (*n*=7 per group, except C26-F + NA *Il6 n*=6, C26-F + NA *Inhba n*=6). Statistical analysis was performed either with two-tailed Student's t-test or ANOVA + Fisher's LSD for normally distributed data and with Kruskal-Wallis + Uncorrected Dunn's test for non-normal data. **k** Kaplan-Meier plot showing fraction of surviving mice in each time point after tumor inoculation. Dotted lines indicate Folfox treatment. Statistical analysis was performed with Mantel-Cox test (C26-F *n*=12, C26-F + NA *n*=11). Data display: **a-d, f, h-j** are means with individual values; **e, g** are means ± SEM; **k** is plotted as a fraction of surviving mice in each group. Original raw data are provided as a Source Data file. NA; niacin, A.U.; arbitrary units.

Supplementary Fig. 4

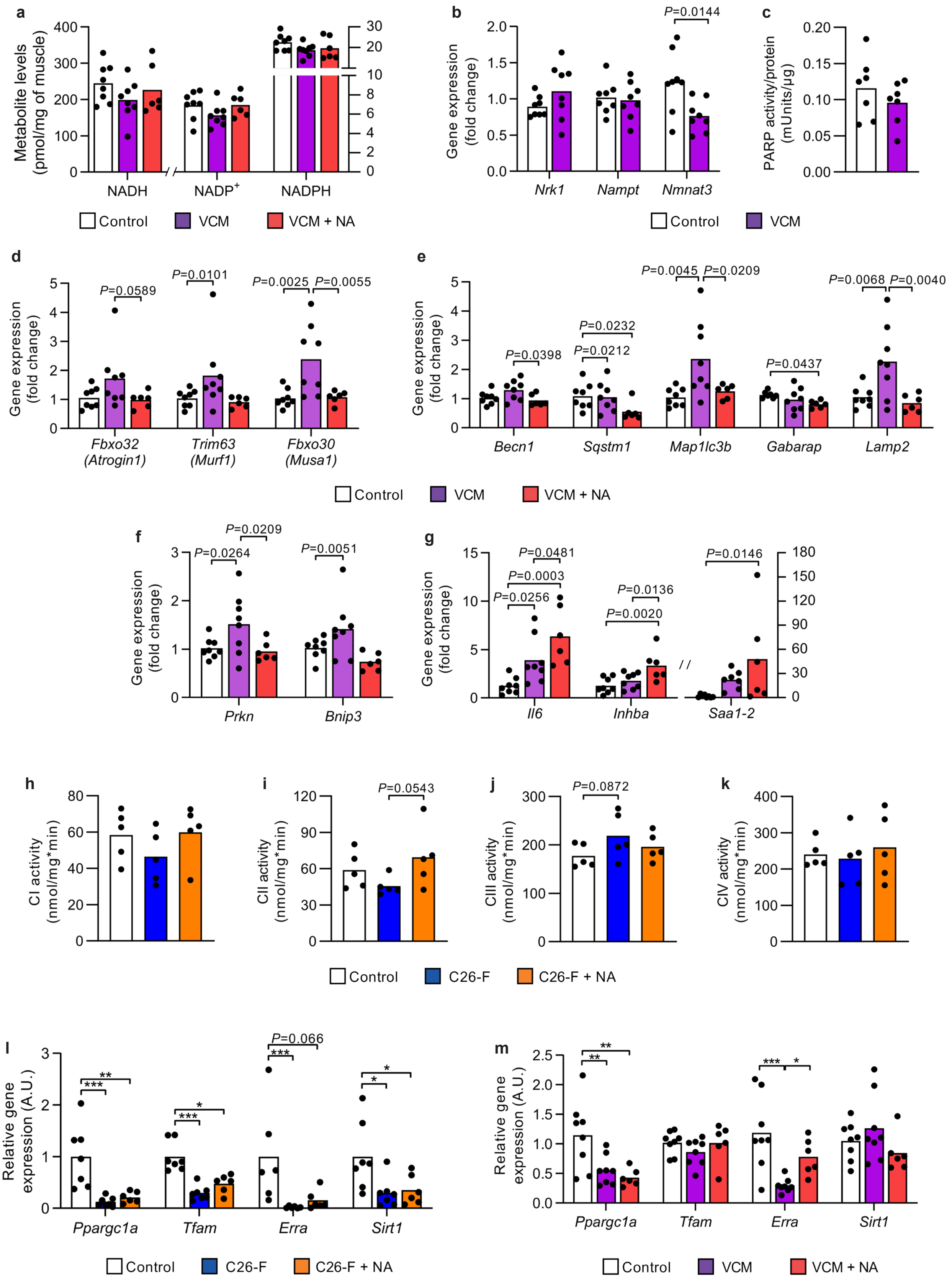

Supplementary Fig. 4. Study of niacin effects in the muscle of C26-F and VCM mice.

a Levels of NADH, $NADP^+$ and NADPH represented as pmol per mg of muscle (control *n*=8, VCM *n*=8, VCM + NA *n*=6). **b** Relative expression of genes involved in NAD^+ biosynthesis in the skeletal muscle (*n*=8 per group). **c** Total PARP activity as mUnits normalized to total protein content (*n*=7 per group). **d-f** Relative expression of E3 ubiquitin ligases (**d**), autophagy (**e**) and mitophagy (**f**) genes in the skeletal muscle (control *n*=8, VCM *n*=8, VCM + NA *n*=6, except Control *Gabarap n*=6). **g** Relative gene expression of *Il6*, *Inhba* and *Saa1-2* genes in the liver of Control, VCM and VCM + NA mice (*n*=8 per group, except VCM *Saa1-2 n*=7). **h-k** Enzymatic activity of mitochondrial complexes (C)I, II, III and IV measured by substrate consumption/product generation over time (nmol/min) normalized by the total amount of protein (mg) in the lysate (*n*=5 per group). **l, m** Relative expression of genes involved in mitochondrial biogenesis in (**l**) C26-F (control *n*=7, C26-F *n*=7, C26-F + NA *n*=6, except control *Erra n*=6, C26-F *Erra n*=6) and (**m**) VCM (control *n*=8, VCM *n*=8, VCM + NA *n*=6) experimental models, respectively. All bar plots display means with individual values. Statistical analysis was performed either with two-tailed Student's t-test or ANOVA + Fisher's LSD for normally distributed data and with Kruskal-Wallis + Uncorrected Dunn's test for non-normal data. Original raw data are provided as a Source Data file. NA; niacin.

Supplementary Fig. 5

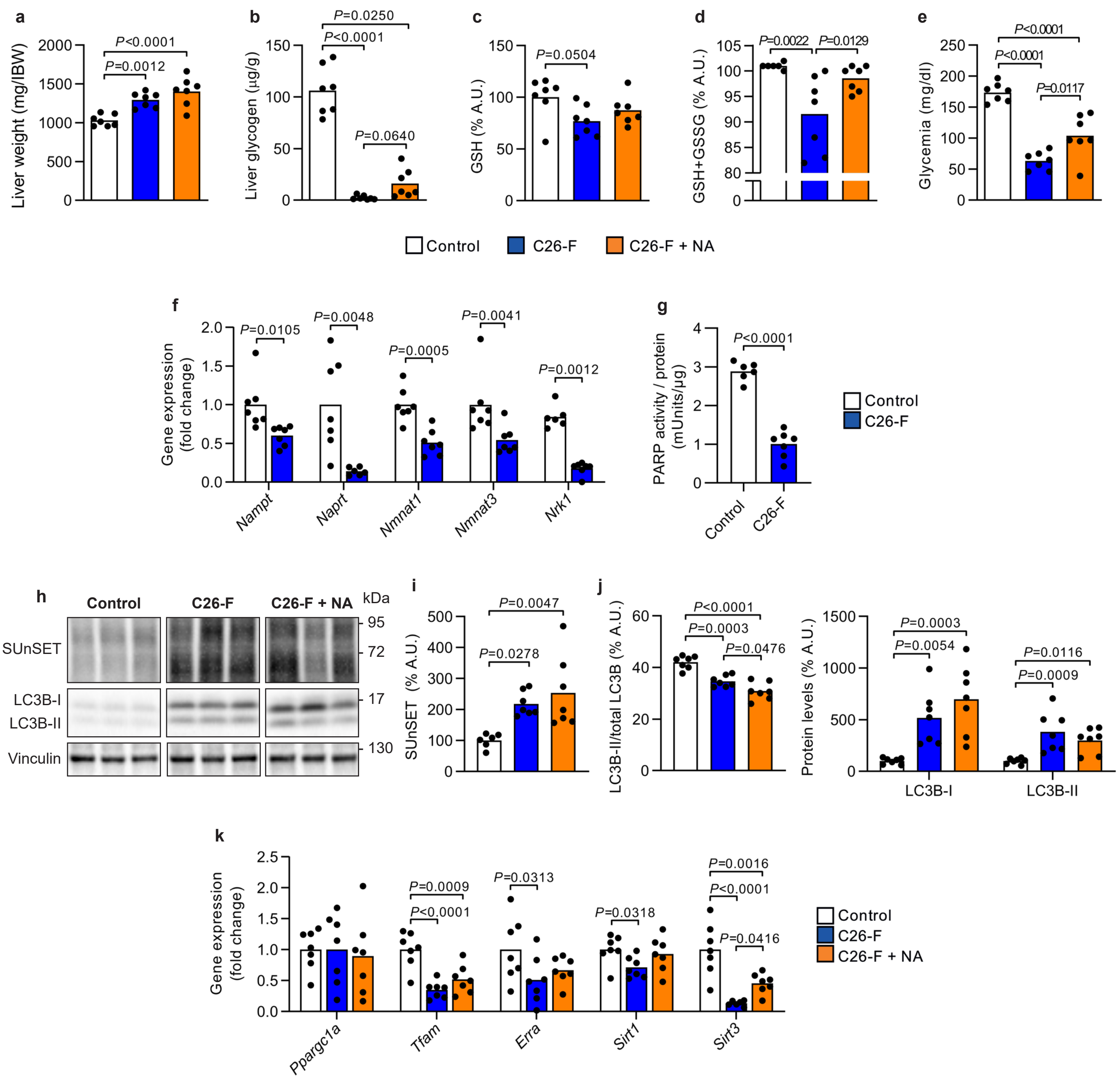

Supplementary Fig. 5. Alterations in NAD⁺ biosynthesis, protein metabolism and mitochondrial homeostasis in the liver of C26-F mice and the impact of niacin treatment.

a Liver wet weight normalized by initial body weight (IBW) from control, C26-F and C26-F + NA groups (*n*=7 per group). **b** Hepatic glycogen content (*n*=7 per group). **c, d** Content of reduced (**c**) and total (**d**) hepatic glutathione (*n*=7 per group, except Control GSH+GSSG *n*=6). **e** Glycemia calculated as mg of glucose per dL of blood (*n*=7 per group). **f** Relative expression of genes involved in NAD⁺ biosynthesis in the liver (*n*=7 per group, except C26-F *Naprt* *n*=6, Control *Nrk1* *n*=6). **g** Total PARP activity as mUnits normalized to total protein content (Control *n*=6, C26-F *n*=7). **h-j** Representative western blotting bands (**h**) and densitometry analysis of puromycin incorporation (SUnSET analysis) (**i**) and LC3B protein (**j**; *n*=7 per group, except control SUnSET *n*=6). Vinculin was used as loading control. **k** Relative expression of genes involved in mitochondrial biogenesis (*n*=7 per group, except C26-F *Sirt3* *n*=6). All bar plots display means with individual values. Statistical analysis was performed either with two-tailed Student's t test or ANOVA + Fisher's LSD for normally distributed data and with two-tailed Mann-Whitney or Kruskal-Wallis + Uncorrected Dunn's test for non-normal data. Original raw data are provided as a Source Data file. NA; niacin.

Supplementary Fig. 6

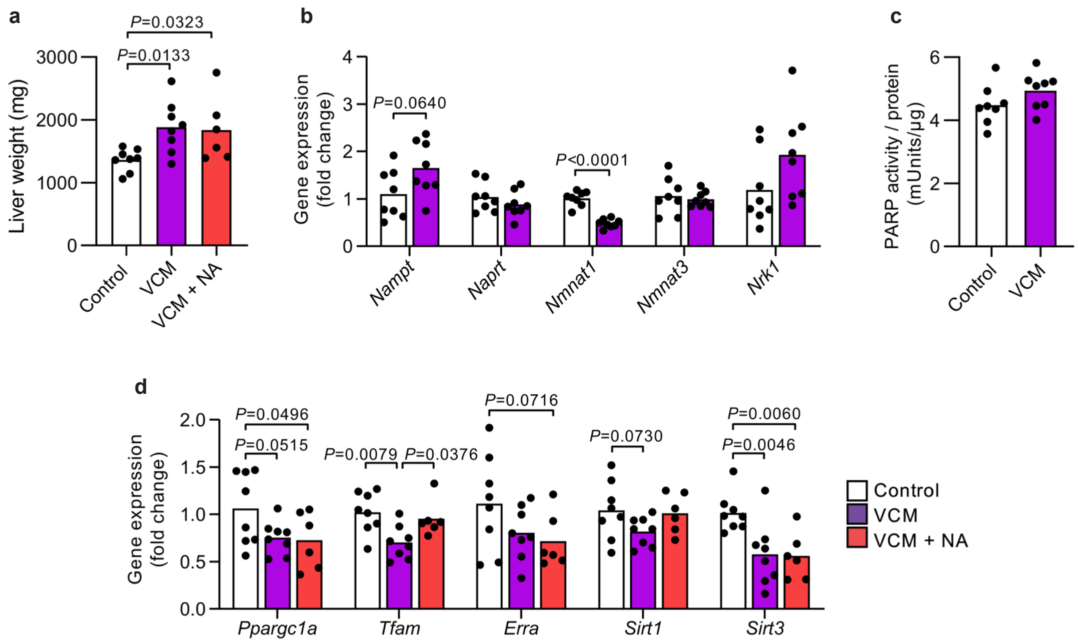

Supplementary Fig. 6. Alterations in NAD$^+$ biosynthesis, protein metabolism and mitochondrial homeostasis in the liver of VCM mice and impact of niacin treatment.

a Liver wet weight from control, VCM and VCM + NA groups (control *n*=8, VCM *n*=8, VCM + NA *n*=6); **b** Relative expression of genes involved in NAD biosynthesis in the liver (*n*=8 per group). **c** Total PARP activity as mUnits normalized to total protein content (*n*=8 per group). **d** Relative expression of genes involved in mitochondrial biogenesis (control *n*=8, VCM *n*=8, VCM + NA *n*=6). All bar plots display means with individual values. Statistical analysis was performed either with two-tailed Student's t test or ANOVA + Fisher's LSD for normally distributed data and with two-tailed Mann-Whitney or Kruskal-Wallis + Uncorrected Dunn's test for non-normal data. Original raw data are provided as a Source Data file. NA; niacin.

Supplementary Table 1. Patient population's characteristics

	Control (*n*=28)	WS (*n*=49)	C (*n*=53)	Total (*n*=130)	*P* value
F % (n)	53.6 (15)	42.9 (21)	52.8 (28)	49.2 (64)	ns
M % (n)	46.4 (13)	57.1 (28)	47.2 (25)	50.8 (66)	
Age, mean years ± SD (Min-Max)	62.7 ± 12.9 (40-86)	68.5 ± 13.1 (40-88)	67.5 ± 9.2 (49-88)		0.02 Cntr vs WS
BMI (kg/m^2), mean ± SD	25.1 ± 3.3[a]	25.6 ± 4.0	23.2 ± 3.6		0.02 (Cntr vs C); 0.0008 (WS vs C)
BMI category (kg/m^2)					0.04
< 20, % (n)	0 (0)	4.1 (2)	17.0 (9)		
20,0 to 24,9, % (n)	59.3 (16)	46.9 (23)	56.6 (30)		
25,0 to 29,9, % (n)	33.3 (9)	34.7 (17)	22.6 (12)		
≥ 30	7.4 (2)	14.3 (7)	3.8 (2)		
Weight loss (%), mean ± SD (Min/Max)	-0.7 ± 2.7 (0/-13)[a]	-0.4 ± 1.0 (0/-4.2)	-11.5 ± 6.0 (-3.1/-26.3)		<0,0001 (Cntr vs C ; WS vs C)
Tumor type, % (n)					
CRC	na	49 (24)	13.2 (7)		0.0002
Pancreas	na	38.8 (19)	77.4 (41)		
Other gastrointestinal	na	12.2 (6)	9.4 (5)		
Drugs, % (n)	50 (11) [b]	61.2 (30)	64.2 (34)		ns
Co-morbidities, % (n)	13.6 (3) [b]	22.4 (11)	28.3 (15)		ns
CT, % (n)	na	14.3 (7)	13.2 (7)		ns
CT < 4 weeks, % (n)	na	6.1 (3)	3.8 (2)		ns
CT > 4 weeks, % (n)	na	8.2 (4)	9.4 (5)		ns

All demographic and clinical data were collected at the time of surgery. Differences between groups were analyzed by one-way ANOVA (continuous variable that passed the normality test) or Kruskal-Wallis test (continuous variable that didn't pass the normality test) with Benjamini, Krieger and Yekutieli adjustment and χ^2 test (categorical variables).

WS, weight-stable; C, cachectic; F, female; M, male; ns, not significant; BMI, body mass index calculated as patient weight (kg)/height (m)^2. Weight loss in 6 months before biopsies were calculated with the following formula: [(current weight [kg] - weight 6 months ago [kg])/weight 6 months ago (kg)] X 100, negative values indicate weight loss; na, not applicable; CRC, colorectal cancer. Other gastrointestinal: duodenum, ampulla of Vater, distal bile duct, anus. Drugs and co-morbidities having an impact on skeletal muscle homeostasis are considered. CT, chemotherapy. CT < 4 weeks, chemotherapy exposure within 4 weeks prior to muscle biopsy. CT > 4 weeks, chemotherapy exposure concluded more that 4 weeks previous to biopsy collection.

[a] Control patients with BMI and weight loss information: *n*=27

[b] Control patients with information on current medication and co-morbidities: *n*=22

-

Supplementary Table 2. Metabolomic patient subset's characteristics

	Healthy (*n*=10)	High NRK2 (*n*=10)	Low NRK2 (*n*=10)	Total (*n*=30)	*P* value
F % (n)	50 (5)	50 (5)	50 (5)	50 (15)	ns
M % (n)	50 (5)	50 (5)	50 (5)	50 (15)	
Age, mean years ± SD (Min-Max)	64.8 ± 15.2 (40-81)	71.5 ± 8.8 (61-86)	64.1 ± 9.3 (49-76)		ns
BMI (kg/m²), mean ± SD	25.2 ± 2,5	22.3 ± 2.6	22.8 ± 3.1		0.04 (Healthy vs High); 0.08 (Healthy vs Low)
BMI category (kg/m²)					ns
< 20, % (n)	0 (0)	20 (2)	30 (3)		
20,0 to 24,9, % (n)	60 (6)	70 (7)	40 (4)		
25,0 to 29,9, % (n)	40 (4)	10 (1)	30 (3)		
≥ 30	0 (0)	0 (0)	0 (0)		
Weight loss (%), mean ± SD (Min/Max)	0 ± 0 (0/0)	-7.5 ± 10.1 (0/-26.3)	-9.7 ± 7.9 (0/-22.4)		0.04 (Healthy vs High); 0.002 (Healthy vs Low)
Cachectic, % (n)	na	40 (4)	70 (7)		ns
Low muscle mass, % (n)	na	60 (6)	50 (5)		ns
Myosteatosis, % (n)	na	50 (5)	30 (3)		ns
Tumor type, % (n)					0.04
CRC	na	20 (2)	0 (0)		
Pancreas	na	80 (8)	60 (6)		
Other gastrointestinal	na	0 (0)	40 (4)		
Tumor stage, % (n)					ns
0	na	10 (1)	10 (1)		
I	na	0 (0)	10 (1)		
II	na	50 (5)	10 (1)		
III	na	10 (1)	50 (5)		
IV	na	30 (3)	20 (2)		
Drugs, % (n)	70 (7)	40 (4)	20 (2)		ns
Co-morbidities, % (n)	10 (1)	10 (1)	10 (1)		ns
CT, % (n)	na	30 (3)	0 (0)		ns
CT < 4 weeks, % (n)	na	20 (2)	0 (0)		ns
CT > 4 weeks, % (n)	na	10 (1)	0 (0)		ns

All demographic and clinical data were collected at the time of surgery. CT scans for body composition analysis were collected within 60 days before surgery.

Cancer patients are classified having low muscle mass in case of SMI<41 cm^2/m^2 in females; SMI<43 cm^2/m^2 if BMI <25, and SMI<53 cm^2/m^2 if BMI ≥25 in males and having myosteatosis in case of HU <33 if BMI ≥25, and <41 if BMI <25, regardless the sex (Martin L. *et al*.). Skeletal muscle index (SMI) was calculated as lumbar total muscle cross-sectional area (cm^2)/height $(m)^2$. Differences between groups were analyzed by one-way ANOVA (continuous variable that passed the normality test) or Kruskal-Wallis test (continuous variable that didn't pass the normality test) with Benjamini, Krieger and Yekutieli adjustment and χ^2 test or Fisher's exact test (categorical variables). F, female; M, male; ns, not significant; BMI, body mass index calculated as patient weight (kg)/height $(m)^2$. Weight loss in 6 months before biopsies were calculated with the following formula: [(current weight [kg] - weight 6 months ago [kg])/weight 6 months ago (kg)] X 100, negative values indicate weight loss; na, not applicable; CRC, colorectal cancer. Other gastrointestinal: duodenum, ampulla of Vater, distal bile duct. Drugs and co-morbidities having an impact on skeletal muscle homeostasis are considered. CT, chemotherapy. CT < 4 weeks, chemotherapy exposure within 4 weeks prior to muscle biopsy. CT > 4 weeks, chemotherapy exposure concluded more that 4 weeks previous to biopsy collection.

Supplementary Table 3. Primers used for qPCR analyses

Species	Gene	Forward	Reverse
mouse	16s (mtDNA)	CCGCAAGGGAAAGATGAAAGAC	TCGTTTGGTTTCGGGGTTTC
mouse	*36b4*	GGCCCTGCACTCTCGCTTTC	TGCCAGGACGCGCTTGT
mouse	*Becn1*	TGAAATCAATGCTGCCTGGG	CCAGAACAGTATAACGGCAACTCC
mouse	*Beta-actin*	CTGGCTCCTAGCACCATGAAGAT	GGTGGACAGTGAGGCCAGGAT
mouse	*Bnip3*	GTCGCCTGGCCTCAGAAC	CCCATTGCCATTGCTGAAGT
mouse	Cox2 (mtDNA)	GTTGATAACCGAGTCGTTCTGC	CCTGGGATGGCATCAGTTTT
mouse	*Erra*	ACTGCCACTGCAGGATGAG	CACAGCCTCAGCATCTTCAA
mouse	*Fbxo30/Musa1*	TCGTGGAATGGTAATCTTGC	CCTCCCGTTTCTCTATCACG
mouse	*Fbxo32/Atrogin1*	GCAAACACTGCCACATTCTCTC	CTTGAGGGGAAAGTGAGACG
mouse	*Gabarap*	TCCGTGCTGAAGATGCCTTG	TCTTCTTCATGGTGTTCCTGGTA
mouse	*H2bc4*	TACAACAAGCGCTCGACCA	TCTGCTCCTCTTGGCAGG
mouse	Hk2 (gDNA)	GCCAGCCTCTCCTGATTTTAGTGT	GGGAACACAAAAGACCTCTTCTGG
mouse	*Hprt*	GAGGAGTCCTGTTGATGTTGCCAG	GGCTGGCCTATAGGCTCATAGTGC
mouse	*Il6*	CTGATGCTGGTGACAACCAC	CAGAATTGCCACATTGCACAAC
mouse	*Inhba*	GAACGGGTATGTGGAGATAG	TGAAATAGACGGATGGTGAC
mouse	*Lamp2*	GCTGAACAACAGCCAAATTA	CTGAGCCATTAGCCAAATACAT
mouse	*Map1lc3b*	CACTGCTCTGTCTTGTGTA	TCGTTGTGCCTTTATTAGTG
mouse	*Nampt*	GAACAGATACTGTGGCGGGA	CCAAGCCGTTATGGTACTGTG
mouse	*Naprt*	GCAGGACTGTATGCGCTTTCT	GAAGCGGCACACCAGGGA
mouse	*Nmnat1*	TGTGCCCAAGGTGAAATTGCT	CCACGATTTGCGTGATGTCC
mouse	*Nmnat3*	CACGAATATGCACCTGCGCT	CATTGACGGGTGAGATGATGC
mouse	*Nrk1*	CCCAACTGCAGCGTCATATC	CCTTGAGCACTTTCCAAGGC
mouse	*Nrk2*	CACCTCAGGACCAGTCACCT	CTGTTGGTCAGGGTGGTCTT
mouse	*Ppargc1a (1)*	AAGTGTGGAACTCTCTGGAACTG	GGGTTATCTTGGTTGGCTTTATG
mouse	*Ppargc1a (2)*	GCAACATGCTCAAGCCAAAC	TGCAGTTCCAGAGAGTTCCA
mouse	*Prkn*	CGTGTGATTTTTGCCGGGAAG	GGTCCACTCGTGTCAAGCTC
mouse	*Saa1-2*	AGTCTGGGCTGCTGAGAAAATC	AATTGGGGTCTTTGCCACTG
mouse	*Sirt1*	GTCTCCTGTGGGATTCCTGA	ACACAGAGACGGCTGGAACT
mouse	*Sirt3*	CTGAAACCGGATGGCGTTTG	ACCATGACCACCACCCTACT
mouse	*Sqstm1*	GGCCACCTCTCTGATAGCTTCT	GACATTGGGATCTTCTGGTGGA
mouse	*Tbp*	TGCACAGGAGCCAAGAGTGAA	CACATCACAGCTCCCCACCA
mouse	*Tfam*	AAGTGTTTTTCCAGCATGGG	GGCTGCAATTTTCCTAACCA
mouse	*Trim63/Murf1*	GGGCCATTGACTTTGGGACA	TCTCCTTCTTCATTGGTGTTCTTCT
mouse	Ucp2 (gDNA)	CTACAGATGTGGTAAAGGTCCGC	GCAATGGTCTTGTAGGCTTCG
human	*ACTB*	GGGAAATCGTGCGTGACA	GGACTCCATGCCCAGGA
human	*NRK2*	AGGATGACTTCTTCAAGCCCC	GGGCACGGGCAAACTTCT
