# Supplementary material for: NAD+ repletion with niacin counteracts cancer cachexia
Source: Nat Commun. 2023 Apr 3;14:1849. doi: 10.1038/s41467-023-37595-6 (PMC10070388; doi:10.1038/s41467-023-37595-6)
Supplement: Supplementary file 3 — Reporting Summary [file 41467_2023_37595_MOESM3_ESM.pdf]

nature portfolio

Corresponding author(s): Roberta Sartori
Eija Pirinen
Fabio Penna

Last updated by author(s): Feb 26, 2023

Reporting Summary

Nature Portfolio wishes to improve the reproducibility of the work that we publish. This form provides structure for consistency and transparency in reporting. For further information on Nature Portfolio policies, see our Editorial Policies and the Editorial Policy Checklist.

Statistics

For all statistical analyses, confirm that the following items are present in the figure legend, table legend, main text, or Methods section.

n/a	Confirmed	
☐	☒	The exact sample size (n) for each experimental group/condition, given as a discrete number and unit of measurement
☐	☒	A statement on whether measurements were taken from distinct samples or whether the same sample was measured repeatedly
☐	☒	The statistical test(s) used AND whether they are one- or two-sided<br>*Only common tests should be described solely by name; describe more complex techniques in the Methods section.*
☐	☒	A description of all covariates tested
☐	☒	A description of any assumptions or corrections, such as tests of normality and adjustment for multiple comparisons
☐	☒	A full description of the statistical parameters including central tendency (e.g. means) or other basic estimates (e.g. regression coefficient) AND variation (e.g. standard deviation) or associated estimates of uncertainty (e.g. confidence intervals)
☐	☒	For null hypothesis testing, the test statistic (e.g. F, t, r) with confidence intervals, effect sizes, degrees of freedom and P value noted<br>*Give P values as exact values whenever suitable.*
☒	☐	For Bayesian analysis, information on the choice of priors and Markov chain Monte Carlo settings
☒	☐	For hierarchical and complex designs, identification of the appropriate level for tests and full reporting of outcomes
☒	☐	Estimates of effect sizes (e.g. Cohen's d, Pearson's r), indicating how they were calculated

Our web collection on statistics for biologists contains articles on many of the points above.

Software and code

Policy information about availability of computer code

Data collection	Generic data: Microsoft Excel (Microsoft Office Professional Plus 2019), qPCR data: Bio-Rad CFX Maestro 1.0 (4.0.2325.0418), Western blotting densitometry: Image Lab Software for PC Version 6.1.
Data analysis	Prism (version 9, GraphPad)

For manuscripts utilizing custom algorithms or software that are central to the research but not yet described in published literature, software must be made available to editors and reviewers. We strongly encourage code deposition in a community repository (e.g. GitHub). See the Nature Portfolio guidelines for submitting code & software for further information.

Data

Policy information about availability of data

All manuscripts must include a data availability statement. This statement should provide the following information, where applicable:
- Accession codes, unique identifiers, or web links for publicly available datasets
- A description of any restrictions on data availability
- For clinical datasets or third party data, please ensure that the statement adheres to our policy

Metabolome raw data are included as a Supplementary Data file. Source data are provided with this paper.

Human research participants

Policy information about studies involving human research participants and Sex and Gender in Research.

Reporting on sex and gender	The patient population's characteristics reported in Table 1 includes the sex of the partecipants. The cohort studied includes both sexes and is balanced (64 females, 66 males, divided in 3 subgroups). Sex was determined based on self-reporting. The analysis was performed in an aggregated form in order to obtain a suffient numerosity and a consequent statistical significance of the results. In a follow-up study, given the currently ongoing enrollment, a sex-based analysis will be `performed.
Population characteristics	The only relevant covariate characteristic was the age of the control group differing as compared to the pre-cachectic group (62.7 ± 12.9 vs 68.0 ± 13.3; P= 0.04). The cachectic group did not differ from either the control nor the pre-cachectic group. Information on drug use and co-morbidities was collected and did not show any difference among the groups.
Recruitment	From 2015 to 2020 consecutive patients with colorectal or pancreatic cancer and control patients undergoing surgery for benign diseases were enrolled at the 3rd Surgical Clinic of the University Hospital of Padova. No selection bias is present, since the sequentiality of medical access was the only criterion.
Ethics oversight	The research project was approved by the Ethical Committee for Clinical Experimentation of Padova (protocol number 3674/AO/15).

Note that full information on the approval of the study protocol must also be provided in the manuscript.

Field-specific reporting

Please select the one below that is the best fit for your research. If you are not sure, read the appropriate sections before making your selection.

☒ Life sciences ☐ Behavioural & social sciences ☐ Ecological, evolutionary & environmental sciences

For a reference copy of the document with all sections, see nature.com/documents/nr-reporting-summary-flat.pdf

Life sciences study design

All studies must disclose on these points even when the disclosure is negative.

Sample size	Sample sizes were calculated using size power analysis methods for a prior determination, on the basis of the SD and effect size previously obtained using the experimental methods used in the study. With a type I error of 0.05 and a power of 0.80, we calculated the minimal sample size for each group to be at least six mice. Considering a likely drop-off effect of 10-20%, we set sample size for each group to eight mice. To reduce the SD, we minimized physiological variation using mice of the same sex and same age.
Data exclusions	Outliers were identified using ROUT (C1=1%) and excluded from the analysis.
Replication	For ethical reasons (3Rs principles of animal experimentation), animal experiments were not repeated, unless performed with a different outcome/endpoint. The survival experiment in C26-F mice confirmed that niacin improves macroscopically animal health, even if the survival, likely depending on tumor progression, did not.
Randomization	In the animal studies, all the mice were randomized and allocated to either the vehicle or the niacin treatment. In the human study, being observational, the allocation was based on internationally accepted diagnostic criteria.
Blinding	The investigators were not blinded given their involvement in both group allocation and data collection / analysis. In an ideal condition, blinding is desirable in every research experiment. Unfortunately, the current study was performed in three independent small research units, therefore blinding would have required the inclusion of people with inedaquate skills with potential technical errors that would have negatively impacted on the quality of the results. Moreover, the experimental work performed in the three independent labs producing consistent results partially supports the fairness of the analyses.

Reporting for specific materials, systems and methods

We require information from authors about some types of materials, experimental systems and methods used in many studies. Here, indicate whether each material, system or method listed is relevant to your study. If you are not sure if a list item applies to your research, read the appropriate section before selecting a response.

Materials & experimental systems

n/a	Involved in the study	
☐	☒	Antibodies
☐	☒	Eukaryotic cell lines
☒	☐	Palaeontology and archaeology
☐	☒	Animals and other organisms
☐	☒	Clinical data
☒	☐	Dual use research of concern

Methods

n/a	Involved in the study	
☒	☐	ChIP-seq
☒	☐	Flow cytometry
☒	☐	MRI-based neuroimaging

Antibodies

Antibodies used

AMPK (1:1000, 07-181, Millipore, polyclonal), p-AMPKThr172 (1:1000, #2535, Cell Signaling, clone 40H9), GAPDH (1:10000, G8795, Merck Sigma-Aldrich, clone GAPDH-71.1), LC3B (1:1000, L7543, Merck Sigma-Aldrich, polyclonal), NRK2 (1:1000, produced in Dr. Gareth G Lavery's lab, polyclonal), OXPHOS Antibody Cocktail (1:1000, ab110413, Abcam), PGC-1α (1:1000, AB3242, Merck Millipore, polyclonal), PINK1 (1:500, SAB2500794, Merck Sigma-Aldrich, polyclonal), Puromycin (1:1000, EQ0001, Kerafast, clone 3RH11), TOMM20 (1:1000, ab186735, Abcam, clone EPR15581-54), Vinculin (1:2000, sc73614, Santa Cruz Biotechnology, clone 7F9). Goat anti-mouse, goat anti-rabbit and rabbit anti-goat HRP-conjugated IgGs (1:8000, Bio-Rad). Mouse TrueBlot ULTRA: Anti-Mouse Ig HRP (1:1000, 18-8817-30, Kerafast, clone eB144).

Validation

AMPK (07-181, Millipore): western blot analysis on rat liver cell lysate.

p-AMPKThr172 (#2535, Cell Signaling): reactivity was determined by western blot analysis on C2C12 cells untreated or treated with oligomycin (0.5 µM).

GAPDH (G8795, Merck Sigma-Aldrich): cell line (HeLa, JURKAT, COS7, NIH-3T3, PC-12, RAT2, CHO, MDBK, MDCK) lysates were separated on SDS-PAGE and proved with 1 µg/mL Monoclonal Anti-GAPDH Clone: GAPDH-71.1

LC3B (L7543, Merck Sigma-Aldrich): whole extracts of human HEK-293T cells (non-treated or treated with tunicamycin for 48 h) were separated on SDS-PAGE and blotted.

NRK2: the validation was performed by the researcher that produced the antibody and published on PMID: 28752046

XPHOS Antibody Cocktail (ab110413, Abcam): western blot analysis on isolated mitochondria from mice brain.

PGC-1α (AB3242, Merck Millipore): Evaluated by Western Blotting in mouse BAT extracts. Western Blotting Analysis: 1:1,000 dilution of this antibody detected recombinant mouse PGC-1, as well as endogenous PGC-1 in 15 µg of nuclear extract and whole cell lysate from mouse brown adipose tissue (BAT). Immunogen peptide blocking completely blocked target band detection. Validation was also performed by the authors using mice with muscle-specific overexpression (PMID: 36388980).

PINK1 (SAB2500794, Merck Sigma-Aldrich): western blot analysis on rat testis lysate (35 µg protein in RIPA buffer). Primary incubation was 1 hour. Detected by chemiluminescence.

Puromycin (EQ0001, Kerafast): C2C12 myoblasts were starved of serum and leucine for 2 hr and then IGF-1 and leucine were added to the medium of some of the cells for 45 min. Puromycin (1uM) was added to the medium of some of the cells (lanes 3-6) 30 min before harvest. Puromycin incorporation was analyzied by western blot.

TOMM20 (ab186735, Abcam): western blot analysis on PC-12 (rat) and NIH/3T3 (mouse) cell lines.

Vinculin (sc73614, Santa Cruz Biotechnology): direct near-infrared western blot analysis of vinculin expression in K-562, NIH/3T3, NTERA-2 cl.D1, HeLa and C2C12 whole cell lysates.

Eukaryotic cell lines

Policy information about cell lines and Sex and Gender in Research

Cell line source(s)

C26 carcinoma cells were a gift from Prof. Mario P. Colombo (Istituto di Ricovero e Cura a Carattere Scientifico National Cancer Institute, Milano, Italy) and used to induce experimental cachexia. The in vivo use of these cells is well established (doi: 10.1016/j.semcdb.2015.09.002.) and adoped in our lab in the last 15 years. C26 cells are not commercially available. Prof. MP Colombo obtained the cells from the laboratory that originated the clone in 1975 (PMID: 1149045)

Authentication

No authentication method was used.

Mycoplasma contamination

C26 cells tested negative for mycoplasma contamination.

Commonly misidentified lines
(See ICLAC register)

Not used.

Animals and other research organisms

Policy information about studies involving animals; ARRIVE guidelines recommended for reporting animal research, and Sex and Gender in Research

Laboratory animals	The animals were maintained on a regular dark-light cycle of 12:12 hours with controlled temperature (20-23°C), humidity (40-60%) and free access to food (2018 Teklad global 18% protein rodent chow) and water during the whole experimental period. Two distinct experiments were performed in this study:<br>- C26-induced cachexia: 3- and 6-month-old wild-type BALB/c mice.<br>- Spontaneous tumors in VCM mice (background C57BL/6J): B6.Cg-Tg(Vil1-cre)997Gum/J (Villin-Cre) and B6.Cg-Msh2tm2.1Rak/J (Msh2loxP), mice were purchased from The Jackson Laboratory (Bar Harbor, CA, USA) and were crossed to obtain the Villin-Cre/ Msh2loxP/loxP(VCM) offspring that were assigned to the experimental groups at the age of 12 months.
Wild animals	The study did not involve wild animals.
Reporting on sex	C26: Females were used to avoid the fighting characteristic among male cagemates subjected to severe cachexia protocols.<br>VCM: one-year-old male mice were used, as in the initial characterization of the model performed in our colony for this study, a higher incidence of tumor-bearing mice in the male population was found.
Field-collected samples	The study did not involve samples collected from the field.
Ethics oversight	The experimental protocols were approved by the Bioethical Committee of the University of Torino (Torino, Italy) and the Italian Ministry of Health (Aut. Nr. 579/2018-PR).

Note that full information on the approval of the study protocol must also be provided in the manuscript.

Clinical data

Policy information about clinical studies

All manuscripts should comply with the ICMJEguidelines for publication of clinical research and a completedCONSORT checklist must be included with all submissions.

Clinical trial registration	Not available.
Study protocol	The research project can be obtained by the co-last author Roberta Sartori or at the Ethical Committee for Clinical Experimentation of Padova (protocol number 3674/AO/15).
Data collection	Being a follow-up of a published research, the full information can be found here: https://www.science.org/doi/suppl/10.1126/ scitranslmed.aay9592/suppl_file/scitranslmed.aay9592_sm.pdf
Outcomes	Being a follow-up of a published research, the full information can be found here: https://www.science.org/doi/suppl/10.1126/ scitranslmed.aay9592/suppl_file/scitranslmed.aay9592_sm.pdf
