# Supplementary material for: NAD+ repletion with niacin counteracts cancer cachexia
Source: Nat Commun. 2023 Apr 3;14:1849. doi: 10.1038/s41467-023-37595-6 (PMC10070388; doi:10.1038/s41467-023-37595-6)
Supplement: Supplementary file 4 — Description of Additional Supplementary Files [file 41467_2023_37595_MOESM4_ESM.pdf]

Description of Additional Supplementary Files

Supplementary Data 1

Description: Raw data of targeted metabolome profiling in skeletal muscle biopsies from Control, Cancer-Low *NRK2* and Cancer-High *NRK2* donors (*n*=10 per group). Values refer to the integrated area of the peak of the metabolite of interest.
